# Virus purification highlights the high susceptibility of SARS-CoV-2 to a chlorine-based disinfectant, chlorous acid

**DOI:** 10.1371/journal.pone.0288634

**Published:** 2023-07-14

**Authors:** Basirat Mojisola Lawal-Ayinde, Tomoko Morita, Kosuke Oda, Tanuza Nazmul, Miuko Kurose, Toshihito Nomura, Akima Yamamoto, Akifumi Higashiura, Tomoyuki Akita, Junko Tanaka, Isanori Horiuchi, Hisataka Goda, Takemasa Sakaguchi

**Affiliations:** 1 Department of Virology, Graduate School of Biomedical and Health Sciences, Hiroshima University, Hiroshima, Japan; 2 Department of Infectious Diseases, Hiroshima University Hospital, Hiroshima, Japan; 3 Department of Epidemiology, Infectious Disease Control, and Prevention, Graduate School of Biomedical and Health Sciences, Hiroshima University, Hiroshima, Japan; 4 Sankei Co., Ltd., Himeji, Japan; East China Normal University School of Life Sciences, CHINA

## Abstract

Chlorous acid water (HClO_2_) is known for its antimicrobial activity. In this study, we attempted to accurately assess the ability of chlorous acid water to inactivate SARS-CoV-2. When using cell culture supernatants of infected cells as the test virus, the 99% inactivation concentration (IC_99_) for the SARS-CoV-2 D614G variant, as well as the Delta and Omicron variants, was approximately 10ppm of free chlorine concentration with a reaction time of 10 minutes. On the other hand, in experiments using a more purified virus, the IC_99_ of chlorous acid water was 0.41–0.74ppm with a reaction time of 1 minute, showing a strong inactivation capacity over 200 times. With sodium hypochlorite water, the IC_99_ was 0.54ppm, confirming that these chlorine compounds have a potent inactivation effect against SARS-CoV-2. However, it became clear that when using cell culture supernatants of infected cells as the test virus, the effect is masked by impurities such as amino acids contained therein. Also, when proteins (0.5% polypeptone, or 0.3% BSA + 0.3% sheep red blood cells, or 5% FBS) were added to the purified virus, the IC_99_ values became high, ranging from 5.3 to 76ppm with a reaction time of 10 minutes, significantly reducing the effect. However, considering that the usual usage concentration is 200ppm, it was shown that chlorous acid water can still exert sufficient disinfection effects even in the presence of proteins. Further research is needed to confirm the practical applications and effects of chlorous acid water, but it has the potential to be an important tool for preventing the spread of SARS-CoV-2.

## Introduction

The emergence of severe acute respiratory syndrome coronavirus 2 (SARS-CoV-2) has become a global health problem, causing respiratory infection COVID-19 in humans after an incubation period of 2 to 10 days, resulting in severe pneumonia and even death in some patients [1]. Aerosolized SARS-CoV-2 has been found to be viable for up to 3 h, and the virus has been found to be viable on plastic and stainless-steel surfaces for up to 72 h [2]. The virus has also been found to remain more stable and infectious for longer periods in environments with smooth or non-porous surfaces [3, 4]. Human-to-human transmission occurs via droplets, but contact transmission is also said to occur when droplets adhere to the surfaces of objects [5]. Therefore, efficient and safe virus inactivators are needed in public places and medical facilities to prevent further virus spread.

In recent years, various chlorine-based disinfectants, such as chlorine dioxide, sodium hypochlorite, and chlorous acid solutions, have been shown to exhibit antiviral effects against SARS-CoV-2 [6–9]. This is because chlorine ions are potent oxidizing agents that induce denaturation of microbial proteins [10]; however, chlorine ions rapidly lose their antimicrobial activity upon contact with organic matter [11]. Among chlorine-based disinfectants, chlorous acid water (HClO_2_) has also been shown to possess strong antiviral activity [12, 13]. Notably, under conditions with high organic matter, chlorous acid is more stable than the common germicide sodium hypochlorite (NaClO) and has been shown to continuously generate chlorine ions [12]. Therefore, chlorous acid water may be more useful for treating contaminated environmental surfaces, such as those found in hospitals.

Anti-microbial agents based on chlorous acid water have also been developed [14]. The agents have been used to treat trophic bacteria (*Staphylococcus aureus*, *Escherichia coli*, Enterohemorrhagic *Escherichia coli* O157, *Campylobacter*), bacterial spores (*Paenibacillus*, *Bacillus*, *Clostridium difficile*) and fungi (*Candida albicans*) [12, 14]. Chlorous acid water has also been shown to be effective against both enveloped viruses (influenza virus, herpes simplex virus-1) and non-enveloped viruses (feline calicivirus, human rhinovirus, human norovirus) [13, 15]. More recently, it was shown to be effective against SARS-CoV-2 [8]. However, a detailed analysis of its action against SARS-CoV-2 has not been conducted.

The aim of this study was to evaluate the inherent SARS-CoV-2 inactivation activity of chlorous acid water as accurately as possible. To achieve this, the free available chlorine (FAC) in the chlorous acid solution was monitored using an appropriate method [16]. During the course of the experiment, it was found that the efficacy of chlorous acid water was reduced when using the "supernatant of infected culture cells," which is commonly employed in standard virus inactivation tests. In order to accurately assess the effects of chlorous acid water, it was necessary to use highly purified virus particles.

## Materials and methods

### Reagents

Chlorous acid water (Klorus Acid・N barrier) was provided by Sankei Co., Ltd. (Ashikaga, Japan), and sodium hypochlorite solutions (PURELOX-S) were purchased from OYALOX Co., Ltd. (Tokyo, Japan). Sodium thiosulphate and polypeptone (PP) (HIPOLYPEPTON-N) were purchased from Fujifilm Wako Pure Chemicals (Tokyo, Japan). Bovine serum albumin (BSA) was purchased from Katayama Chemical Industries Co., Ltd. (Osaka, Japan), and sheep red blood cells (SRBCs) were purchased from JAPAN BIO SERUM CO., LTD. (Tokyo, Japan). Fetal bovine serum (FBS) was purchased from BIOSERA (Kansas City, MO, USA).

### Measurement of free available chlorine levels

Free available chlorine (FAC) levels of the chlorous acid water and sodium hypochlorite solutions were determined by the DPD method using an RC-V2-CAW meter (Kasahara Rika Kogyo, Saitama, Japan) at the time of use as described previously [16]. The solutions were diluted with sterile distilled water to yield chlorous acid water and sodium hypochlorite solutions with different FAC levels.

### Cell culture

VeroE6/TMPRSS2 cells (African green monkey kidney-derived cells expressing human TMPRSS2; [17] were provided by the Japanese Collection of Research Bioresources (JCRB) Cell Bank (JCRB1819). The cells were maintained in Dulbecco’s Modified Eagle’s Medium (DMEM; Fujifilm Wako Pure Chemicals) containing 10% FBS and 1 mg/mL G418 (Nacalai Tasque, INC., Kyoto, Japan) at 37°C in 5% CO_2_.

### Viruses

The SARS-CoV-2/JP/Hiroshima-46059T/2020 strain (B.1.1, GISAID accession ID: EPI_ISL_6289932), SARS-CoV-2/JP/HiroC77/2021 [Delta (B.1.617.2-like), EPI_ISL_6316561], and SARS-CoV-2/JP/FH-229/2021 [Omicron (BA.1-like), EPI_ISL_11505197], which were all isolated in Hiroshima, were used. For virus stock preparations, the infected cell culture medium was collected at 48 h post-infection, clarified by low-speed centrifugation, and filtrated through a 0.45-μm filter.

Further purification and concentration were performed from the viral stock. For polyethylene glycol (PEG) precipitation, 21 ml of the virus stock was mixed with 1/3 vol (7 ml) of 40% PEG8000 (Promega), placed on ice for 6 h, centrifuged at 3000 rpm for 30 min, and suspended in 1 ml of saline. For ultracentrifugation, 21 ml of the virus stock was centrifuged in a Beckman SW-32Ti at 22000 rpm for 1 h at 4C, and the pellet was suspended in 1 ml of saline. Each virus was divided into aliquots and frozen at -80°C until use.

The virus titer was determined by the standard 50% tissue culture infectious dose (TCID_50_) method and expressed as TCID_50_/ml as described previously [18]. All experiments dealing with infectious SARS-CoV-2, including infection and electron microscope sample preparation, were performed at the BSL3 facility at Hiroshima University.

### SDS-PAGE analysis

Each of the prepared viruses was mixed with an equal volume of 2x SDS sample buffer (50 mM Tris-HCl, pH 6.5, 10% glycerol, 2% SDS and 0.1% bromophenol blue) and heated at 90°C. Samples were separated by electrophoresis on a 15% SDS-PAGE gel prepared with WIDE RANGE Gel Preparation Buffer (NACALAI TESQUE, Kyoto, Japan). After electrophoresis, the gel was stained with Coomassie brilliant blue and visualized with the use of the iBright FL1500 Imaging System (Thermo Fisher Scientific).

### Transmission electron microscopy

For electron microscopic observation, the virus solution (3 μl) was adsorbed on a glow discharged carbon-coated Cu grid (EM Japan, Tokyo, Japan) for 30 seconds and excess solution was removed with filter paper. Then 0.9% (w/v) NaCl was added to the grid for washing and was blotted on filter paper after 30 seconds. Finally, the staining solution [2%(w/v) ammonium molybdate tetrahydrate in water] was plotted on the grid and blotted on filter paper after 30 seconds. The staining process was performed twice, and the grids were air-dried and then irradiated with ultraviolet light (Care222; Ushio Inc., Tokyo, Japan) to completely inactivate the virus [18]. Observation of the sample grids was performed using a transmission electron microscope (JEM-1400, JEOL, Tokyo, Japan) at 80 kV. Images were recorded with an equipped CCD camera (1024 x 1024 pixels).

### Virucidal test

Chlorous acid water and sodium hypochlorite solutions were handled in a polystyrene tube throughout the experiment including mixing with the reagent and virus reaction. The solution (90 μl) was reacted with the viral inoculum (10 μl) for 10 min at room temperature before the addition of 10 μl of 1 M sodium thiosulphate. The reaction solutions were serially diluted in 10-fold steps using 10% FBS-DMEM in a 1.5-ml tube and then inoculated onto VeroE6/TMPRSS2 monolayers in a 96-well plate. The inoculated cells were incubated in a CO_2_ incubator until CPE fully appears. The cytopathic effects were observed under a microscope and the TCID_50_/ml values were calculated by the Behrens-Käber method as described previously [19]. Cytotoxic effects of the solutions were determined by microscopic analysis of the cell monolayer in the absence of the virus.

The following reagents were used in the protein loading experiments. In the experiment with a final concentration of 0.5% polypeptone (PP), a 10% (w/v) aqueous solution was prepared, then sterilized by filtration through a 0.1-μm filter, and mixed with an equal volume of virus solution for use in the experiment. In the mixture with the virus, the concentration of PP was 5% (w/v), and since the virus and reagent were mixed at a ratio of 1:9, the final concentration of PP in the reaction solution was 0.5%. For the test with 0.03% BSA, a 0.6% (w/v) BSA aqueous solution was prepared in a similar way to obtain a final concentration of 0.03% in the reaction solution. For the test with 5%(v/v) FBS, 100% FBS was used to obtain a final concentration of 5%(w/v). In the experiment with a final concentration of [0.3% sheep red blood cells (SRBCs) + 0.3% BSA], the virus solution, (3% SRBCs + 3% BSA), and a disinfectant were mixed in a ratio of 1:1:8. In this case, the concentration of the disinfectant was diluted by eight-ninths of that in the other assays, and it was corrected in the final calculations.

### Analysis of disinfection kinetics

Experimental data were fitted to the Chick-Watson model [15]:

$${Log}_{10}\left[\frac{N(t)}{N_{0}}\right]=-k∙C^{n}∙t.$$ (Eq 1)

In **Eq 1**, *N*(*t*) is the remaining virus infectivity after incubation with the disinfectant, and *N*_0_ is the initial infectivity. *C* is a given concentration of the disinfectant, and *t* is the incubation time with the disinfectant. *k* and *n* are the rate constant for a specific disinfectant and condition, and the dilution coefficient to determine the relative importance of the disinfectant concentration, respectively, and they were estimated by the non-linear least squares method.

## Results

### Inactivation of SARS-CoV-2 in supernatants of infected cells

**Fig 1:**
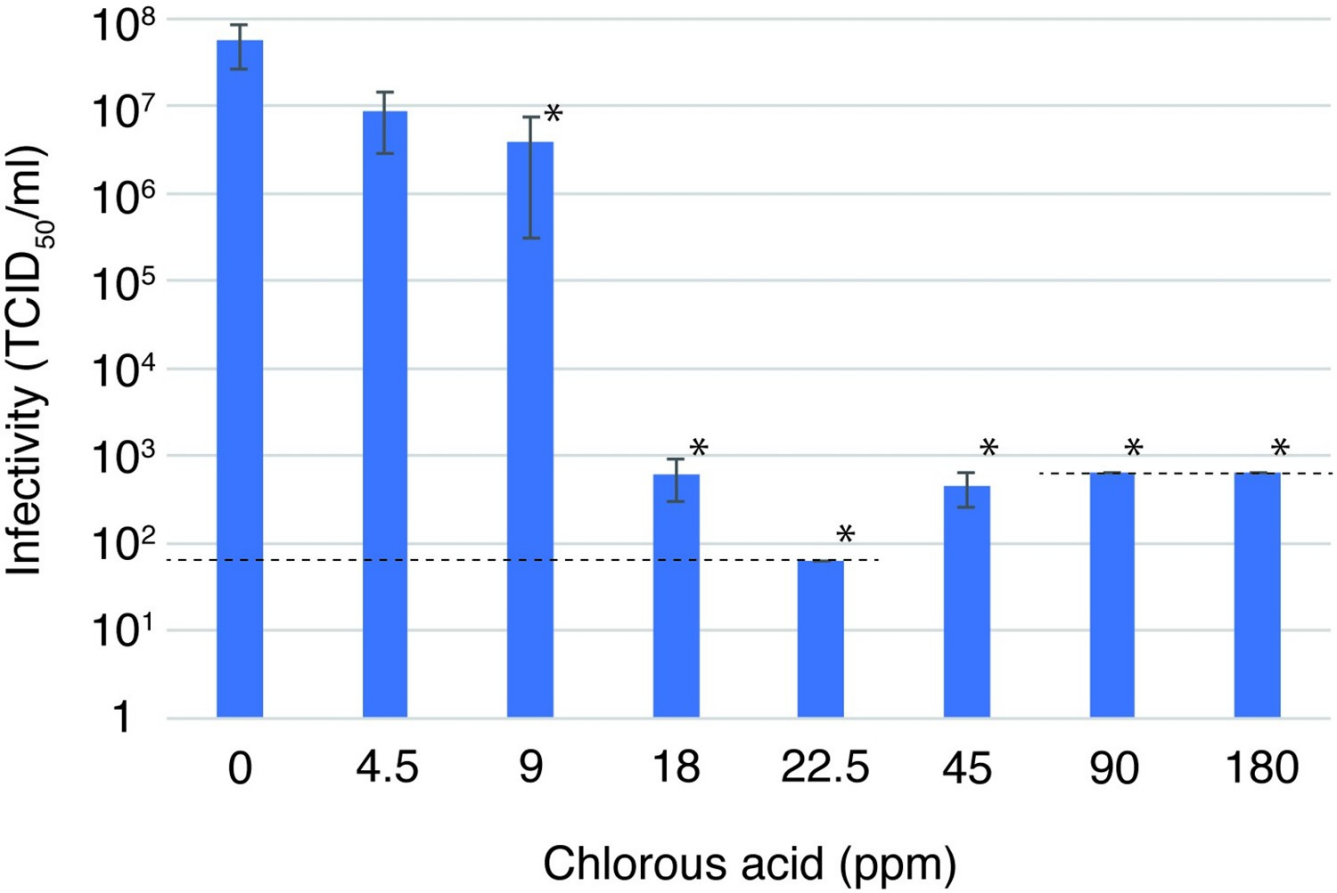
Inactivation of SARS-CoV-2 by chlorous acid water. The virus was incubated with chlorous acid water with different concentrations for 10 min and the infectivity was measured by the TCID_50_ method after quenching chloride ions with sodium thiosulphate. The final concentrations in the reaction solution are shown in the graph. The dotted lines indicate the detection limits of the infectivity assay due to the cytotoxicity of chlorous acid water. Experiments were performed in triplicate, and error bars indicate standard error. *: P < 0.05, Mann-Whitney U test, compared with 0 ppm (water).

VeroE6/TMPRSS2 cells were infected with viruses and cultured with serum-free DMEM, and the culture supernatant was used as the test virus to examine the SARS-CoV-2-inactivating effect of chlorous acid water. The FAC in the chlorous acid water was determined, and the virus was mixed with solutions of various concentrations and allowed to react for 10 minutes. After the reaction was stopped with sodium thiosulfate, the virus infectivity was measured. The results showed that the infectivity titer of the virus decreased to about 1/10 at 9 ppm of FAC in chlorous acid water and significantly decreased to 1/100000 at 18 ppm. It was further reduced to the detection limit at 22.5 ppm, indicating that the virus was almost completely inactivated (Fig 1).

Chlorine concentrations in chlorous acid water shown in Fig 1 and the decreases in infection titer were fitted to an approximation by the Chick-Watson model, which is designed for modeling factors such as chloride ions, the effects of which accumulate over time [20].

**Fig 2:**
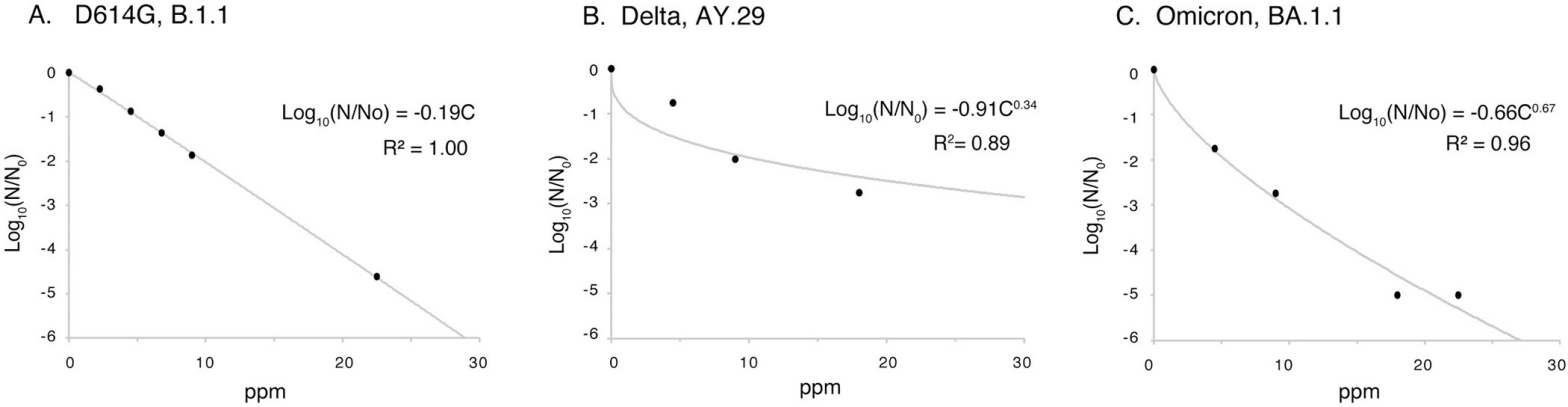
Inactivation of SARS-CoV-2 by chlorous acid water. (A) D614G, Lineage B.1.1, (B) a Delta variant, Lineage AY.29, and (C) an Omicron variant, Lineage BA.1.1. The virus was incubated with chlorous acid water with different concentrations for 10 min and the infectivity was measured by the TCID_50_ method after quenching chloride ions with sodium thiosulphate. Measurements were made and fitted to an approximate equation based on the Chick-Watson model. Approximation equations and R^2^ values are shown in the graphs. R^2^: coefficient of determination, Log_10_(N/N_0_): log reduction in survival ratio.

The coefficients k and n in the equation were calculated, and an approximate curve was drawn (Fig 2A, S1 Table). Based on this approximation, the concentrations of chlorous acid water that reduce infectivity to 50%, 99%, and 99.99% in a 10-minute reaction were derived (Table 1). The concentration at which chlorous acid water reduced the virus to 50% inactivation (IC_50_) was 1.5 ppm, the concentration at which the virus was reduced to 1% (99% inactivation) (IC_99_) was 9.9 ppm, and the concentration at which the virus was reduced to 0.01% (99.99% inactivation) (IC_99.99_) was 19 ppm. This indicates chlorous acid water’s potential for significant SARS-CoV-2 inactivation.

**Table 1:** Inactivation concentrations of SARS-CoV-2 variants with chlorous acid water.

Virus	Inactivation Concentration (ppm)		
	50% Inactivation	99% Inactivation	99.99% Inactivation
	(IC_50_)	(IC_99_)	(IC_99.99_)
D614G, B.1.1	1.5	9.9	19
Delta, AY.29	1.8	12	23
Omicron, BA.1.1	0.31	5.3	14

From Fig 2 and S1 Table, we calculated the concentrations that would inactivate 50%, 99%, or 99.99% of SARS-CoV-2 based on the Chick-Watson model when the SARS-CoV-2 variants were reacted with chlorous acid water for 10 min.

### Effect of chlorous acid water on SARS-CoV-2 Delta and Omicron variants

In the tests conducted thus far, we have been using the so-called European variant D614G (B.1.1) of SARS-CoV-2. However, we also considered the Delta (AY.29) and Omicron (BA.1.1) variants, which have a higher infection transmission efficiency and have been the main strains spreading in Japan to date. The results of infection titers measured with various concentrations of chlorous acid water were fitted to the Chick-Watson model (Fig 2B and 2C, S1 Table). The IC_99_ of chlorous acid water for the Delta variant was 12 ppm, and the IC_99_ of chlorous acid water for the Omicron variant was 5.3 ppm, relatively close to the value of 9.9 ppm for the D614G variant, suggesting that they were equally effective (Table 1).

### Viral purification

In the case of viruses that are prone to budding, like influenza virus and SARS-CoV-2, it is common to use the supernatant of infected cell cultures as the virus solution, which contains amino acids derived from culture media such as DMEM. On the other hand, it has been pointed out that the effect of chlorous acid water on feline calicivirus, poliovirus, and human norovirus is greatly influenced by the co-existing proteins and amino acids [12, 13, 15]. Therefore, to confirm the original effect of chlorous acid on SARS-CoV-2, we purified and concentrated the virus using two methods: the PEG precipitation method and the ultracentrifugation method.

**Fig 3:**
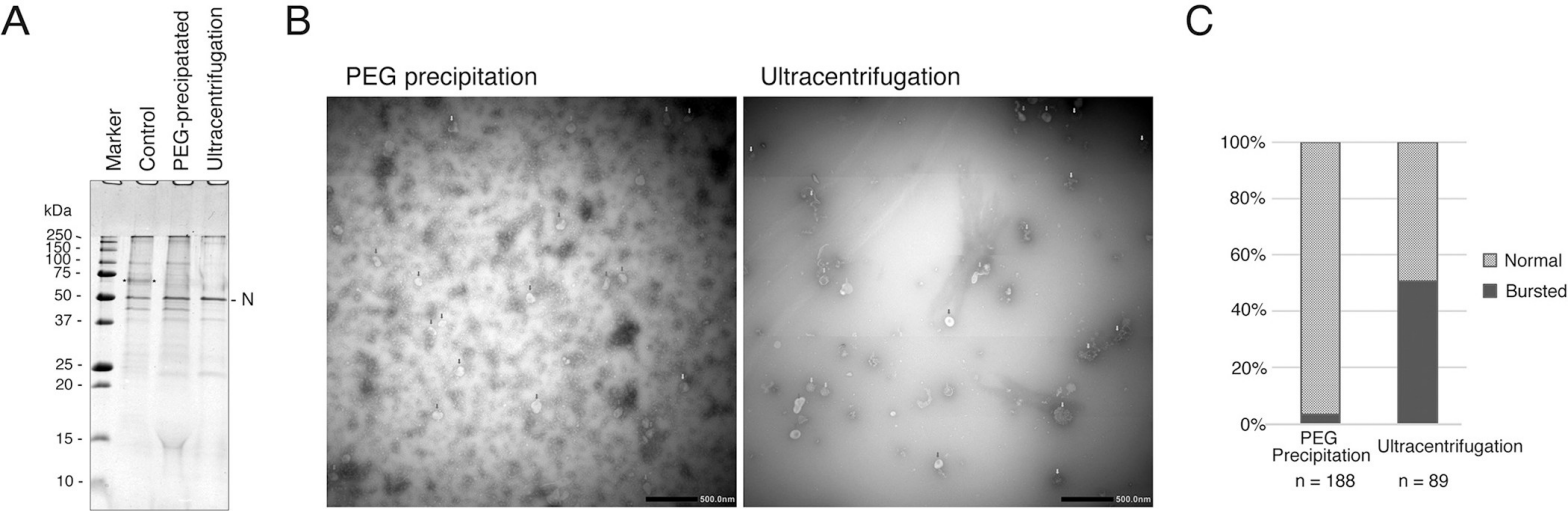
Analysis of PEG-purified and ultracentrifuged viruses. (A) SDS-PAGE analysis. Equivalent amounts of the virus after concentration (concentrated by an Amicon Ultra-15 filter unit, MWCO 100kDa) (lane “control”), the virus precipitated by polyethylene glycol (PEG) precipitation, and the virus sedimented by ultracentrifugation were analyzed by SDS-PAGE and Coomassie brilliant blue staining. The possible major cellular protein band is marked by stars. The position of the SARS-CoV-2 N protein is shown. (B) Electron microscopy. SARS-CoV-2 was purified and concentrated by PEG precipitation or ultracentrifugation, negative stained, and observed by transmission electron microscopy. Red arrows indicate relatively intact viral particles, and yellow arrows indicate viral particles that have been destroyed. (C) Graph showing the ratio of apparent intact and destroyed viruses in B.

SDS-PAGE analysis of the purified virus particles revealed proteins that appeared to be cell-derived (Fig 3A) in the infected supernatant grown in cultured cells and concentrated to the same level by an ultrafiltration membrane unit (Amicon Ultra-15 filter unit, MWCO 100kDa). PEG precipitation eliminated the prominent proteins, but many bands of other cell-derived proteins were observed. Ultracentrifugation appeared to eliminate most of the cellular proteins (Fig 3A).

Electron microscopy (Fig 3B) showed that in the PEG precipitation, some substance other than the virions was visible in the background, but many virus particles were also observed. On the other hand, in the sample purified by ultracentrifugation, little substance other than the virions was visible in the background, but many particles that appeared to have been destroyed were observed. By counting the morphologically intact particles and particles that had been destroyed on the micrographs, we found that about 50% of the virus was destroyed by the ultracentrifugation method. In comparison, only about 3% of the virus was destroyed by PEG precipitation (Fig 3C, S2 Table). While it cannot be said that all of these morphological changes correlate with a decrease in infectivity, it is believed that they are indeed related.

In the case of PEG precipitation, the total infectious titer after enrichment was about half of the titer before enrichment, showing minimal loss of infectious titer due to virus enrichment. In contrast, the ultracentrifugal method reduced the titer to about 1/30 (Table 2).

**Table 2:** Infectivity of PEG-precipitated and ultracentrifuged viruses.

	Infectivity	Volume (ml)	Total infectivity	Ratio of Total infectivity
	(TCID_50_/ml)		(TCID_50_)	
Before concentration	6.3 x10^7^	21	1.3 x 10^9^	1
Concentrated by PEG-precipitation	6.3 x 10^8^	1	6.3 x 10^8^	0.48
	Infectivity	Volume(ml)	Total infectivity	Ratio of Total infectivity
	(TCID_50_/ml)		(TCID_50_)	
Before concentration	6.3 x 10^7^	21	1.3 x 10^9^	1
Concentrated by ultracentrifugation	3.6 x 10^7^	1	3.6 x 10^7^	0.03

SARS-CoV-2 was purified and concentrated by poly ethylene glycol (PEG) precipitation or ultracentrifugation. Infectivity, volume, total infectivity and its ratio are shown in the table.

The above-described results indicate that PEG precipitation does not entirely remove proteins, but the viral infection titer can be concentrated with almost no loss. On the other hand, in the ultracentrifugal method, foreign proteins were almost completely removed, but the virus was severely damaged, and the infection titer was greatly reduced.

### Inactivation of purified SARS-CoV-2 with chlorous acid water

**Fig 4:**
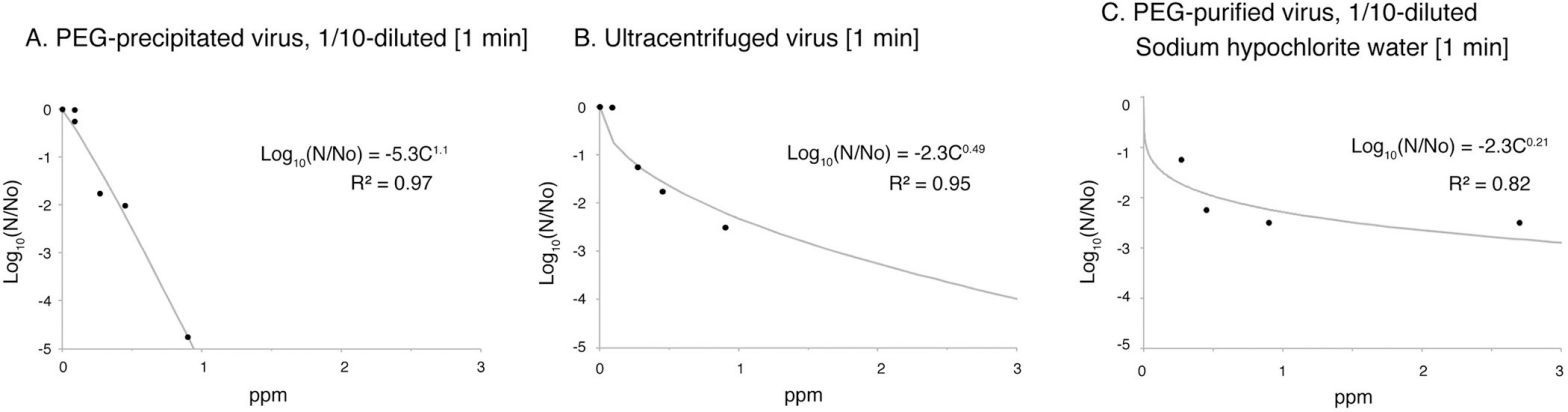
Inactivation of PEG-purified and ultracentrifuged SARS-CoV-2 by chlorine reagents. The 1/10-diluted PEG-purified virus (A) and the ultracentrifuged virus (B) were incubated with chlorous acid water with different concentrations. (C) The 1/10-diluted PEG-precipitated SARS-CoV-2 was incubated with sodium hypochlorite solution. The incubation time of the virus and chlorine reagents was 1 min, and the infectivity was measured by the TCID_50_ method. Measurements were made and fitted to an approximate equation based on the Chick-Watson model. Approximation equations and R^2^ values are shown in the graphs. R^2^: coefficient of determination, Log_10_(N/N_0_): log reduction in survival ratio.

The effects of chlorous acid water on both PEG-purified and ultracentrifuge-purified viruses was examined: PEG-purified viruses had a high infectivity titer, and they were therefore diluted 10-fold with PBS, expecting dilution of any remaining contaminants. The effect of chlorous acid water on these viruses was quite strong, and it was necessary to significantly dilute the reagent, which we thought would make reproducibility difficult. Therefore, we shortened the reaction time from 10 minutes to 1 minute (Fig 4A and 4B, S1 Table). The IC_99_ values of the PEG-purified and ultracentrifuge-purified viruses were calculated to be 0.41 ppm and 0.74 ppm, respectively (Table 3). When the IC_99_ values were recalculated assuming a 10-minute reaction, they were 0.051 ppm and 0.007 ppm, respectively, indicating an approximately 200-fold increase in effectiveness compared to that with the use of the unpurified virus (Table 3). This indicates that chlorous acid water has an extremely powerful ability to inactivate SARS-CoV-2. However, in the case of viruses prepared by conventional methods, the effect of chlorous acid may be weakened by amino acids in the culture medium or foreign substances that appear to be derived from cultured cells. Therefore, it is thought that purifying the virus enables assessment of the intrinsic strong inactivation ability.

**Table 3:** Inactivation concentrations of PEG-purified SARS-CoV-2 with chlorous acid water under protein loading.

Virus	Reagent	Inactivation Concentration (ppm)		
		50% Inactivation	99% Inactivation	99.99% Inactivation
		(IC_50_)	(IC_99_)	(IC_99.99_)
PEG-purified virus, 1/10-diluted	Chlorous acid water	0.073 [1 min]	0.41 [1 min]	0.77 [1 min]
		0.0091 [10 min]	0.051 [10 min]	0.095 [10 min]
Ultracentrifugation-purified virus	Chlorous acid water	0.016 [1 min]	0.74 [1 min]	3.0 [1 min]
PEG-purified virus, 1/10-diluted	Chlorous acid water + 0.03% BSA	3.3 x 10^−5^ [10 min]	0.31 [10 min]	21 [10 min]
PEG-purified virus, 1/10-diluted	Chlorous acid water + 0.3% SRBCs, 0.3% BSA	1.0 [10 min]	5.3 [10 min]	14 [10 min]
PEG-purified virus, 1/10-diluted	Chlorous acid water + 0.5% PP	18 [10 min]	76 [10 min]	128 [10 min]
PEG-purified virus, 1/10-diluted	Chlorous acid water + 5% FBS	7.5 x 10^−3^ [10 min]	11 [10 min]	158 [10 min]
PEG-purified virus, 1/10-diluted	Sodium hypochlorite water	7.7 x 10^−5^ [1 min]	0.54 [1 min]	14 [1 min]

B Based on Fig 4 and S1 Table, we calculated the concentrations that would inactivate 50%, 99%, or 99.99% of 1/10-diluted PEG-purified SARS-CoV-2 based on the Chick-Watson model when the virus was reacted with chlorous acid water with or without protein loading for 10 min. In the case of a one-minute reaction, it is indicated in the table, and the IC_50_, IC_99_, and IC_99.99_ values assuming a 10-minute reaction are shown for the case of 1/10-diluted PEG-purified virus and chlorous acid water. BSA: bovine serum albumin, SRBCs: sheep red blood cells, PP: polypeptone, FBS: fetal bovine serum.

### Effect of sodium hypochlorite on purified SARS-CoV-2

In order to confirm whether the high inactivation effect of chlorous acid water on PEG-purified SARS-CoV-2 is common to chlorine-based disinfectants, we investigated the effect of sodium hypochlorite solution, which is widely used (Fig 4C, S1 Table). Sodium hypochlorite solutions were prepared and tested at matched free available chlorine (FAC) concentrations. As a result, the IC_99_ value of sodium hypochlorite solution for PEG-purified virus was 0.54 ppm after 1-minute exposure, demonstrating a nearly equivalent SARS-CoV-2 inactivation ability to that of chlorous acid water (Table 3). From these findings, it can be inferred that the high virus inactivation effect is due to the action of chlorine ions.

### Effect of chlorous acid water on purified SARS-CoV-2 under the condition of protein loading

To determine whether the coexistence of proteins in the purified viruses affected their inactivation by chlorous acid water, we performed protein-loading experiments using PEG-purified viruses. It is assumed that 0.3% sheep red blood cells (SRBCs) + 0.3% bovine serum albumin (BSA) or 0.03% BSA indicates contamination of the specimen by blood [21] and that 0.5% polypeptone (PP) indicates contamination by vomit [22]. Furthermore, the concentration of protein contained in saliva is 0.67 to 2.37 mg/ml [23], and it was substituted with 5% FBS of equivalent protein concentration [24].

**Fig 5:**
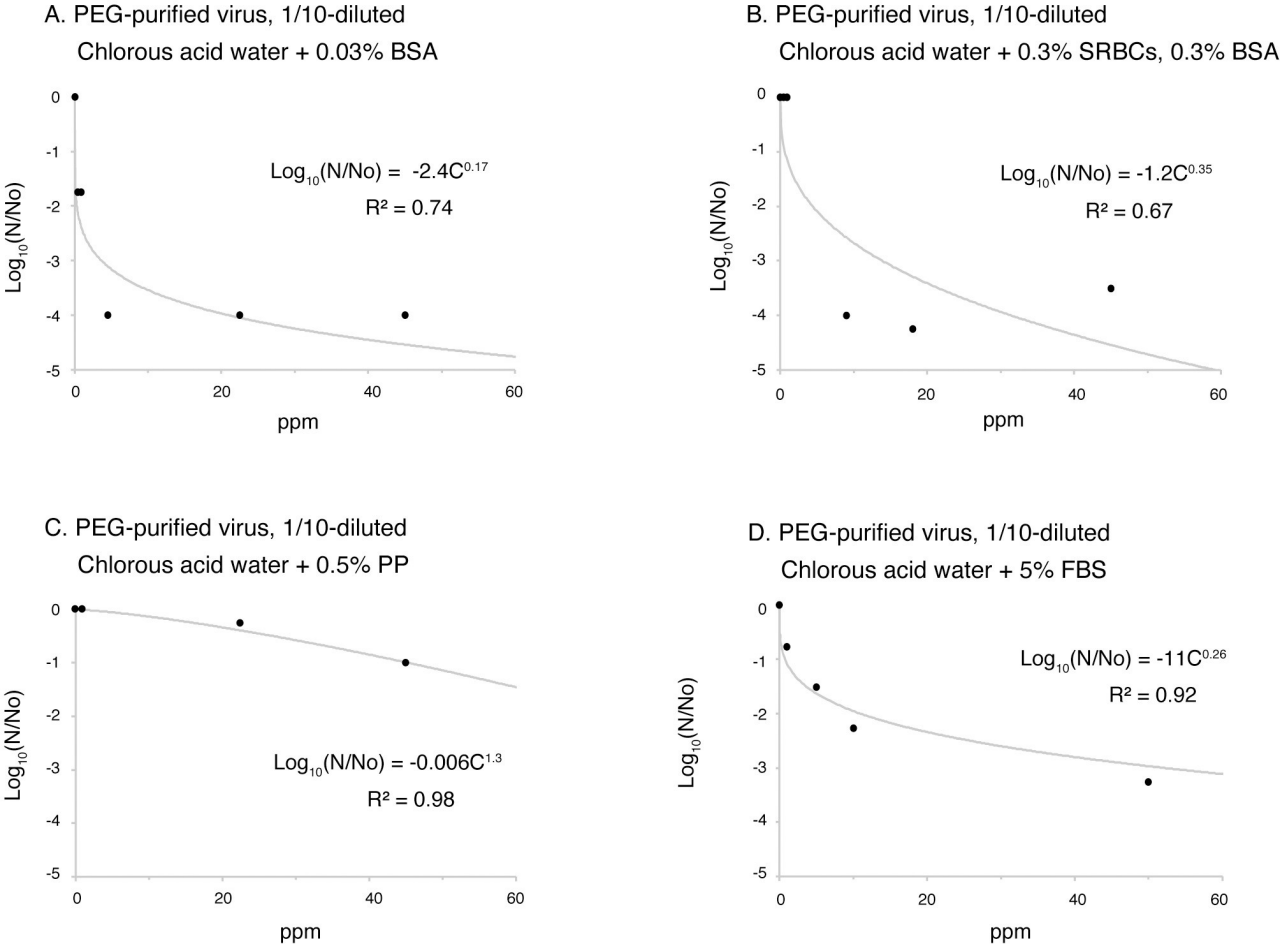
Inactivation of PEG-purified SARS-CoV-2 by chlorous acid water under various protein loads. The 1/10-diluted, PEG-precipitated SARS-CoV-2 was incubated with chlorous acid water along with 0.03% bovine serum albumin (BSA) (A), 0.3% sheep red blood cells (SRBCs) + 0.3% BSA (B), 0.5% polypeptone (PP) (C), and 5% fetal bovine serum (FBS). The incubation time for the virus and the chlorous acid water was 10 minutes, and the infectivity was assessed using the TCID50 method. Measurements were made and then fitted to an approximate equation based on the Chick-Watson model. The approximation equations and R2 values are displayed in the graphs. R2: coefficient of determination, Log10(N/N0): log reduction in the survival ratio.

Approximate curves were prepared for the effect of chlorous acid water on each of the above protein loads, and the inactivation concentrations were calculated (Fig 5, Table 3 and S1 Table). In the case of adding 0.03% BSA, the IC_99_ was 0.31ppm, and for 0.3% SRBCs + 0.3% BSA, the IC_99_ was 5.3ppm, showing an inferior inactivation effect compared to chlorous acid water alone (IC_99_ = 0.051). For 5% FBS, the IC_99_ was 11ppm. When 0.5% PP was added, it was even higher at 76ppm, and was inactivated over 1500 times more strongly than when using chlorous acid water alone (Table 3).

### The pH of the chlorine disinfectants used

The pH could be extremely low or high in both chlorous acid water (HClO_2_) and Na hypochlorite water (NaHClO). Therefore, we measured the pH of these chlorine-based disinfectants used in this study. The pH of chlorous acid water was 5.67 ± 0.11 (mean ± standard error, n = 3) at 20 ppm and 5.53 ± 0.07 at 200 ppm. Meanwhile, the pH of Na hypochlorite water was 7.63 ± 0.02 at 20 ppm and 8.51± 0.04 at 200 ppm. These pH values show that they are acidic and alkaline, respectively.

Excessive acidity and alkalinity, as the pH value is less than 4 or greater than 11, have been pointed out to potentially have significant antiviral effects against SARS-CoV-2 [9]. However, the disinfectants used in this study do not have such extreme pH levels. Therefore, it is considered unlikely that the viral infectious titer would decrease due to pH at relatively short reaction times of one minute or ten minutes.

## Discussion

In this study, we demonstrated the high virucidal ability of chlorous acid water against SARS-CoV-2 using purified and concentrated virus particles. The IC_99_ values of chlorous acid water were 0.41 ppm for PEG-purified virus and 0.74 ppm for ultracentrifugation-purified virus after 1 minute of reaction. Additionally, when using sodium hypochlorite solution with PEG-purified virus, the IC_99_ was 0.54 ppm [1 minute], indicating nearly similar efficacy. Hatanaka et al. [8] reported that the infectivity of SARS-CoV-2 was reduced to about 1/100 with 1 ppm of chlorous acid water and a 10-second reaction. They also used PEG-purified virus, and our study results are in close agreement with their findings, and chlorine ions consistently demonstrate a high inactivation capacity against SARS-CoV-2.

Typically, for viruses like SARS-CoV-2 that readily bud, the supernatant of infected cells is used as the test virus without purification. We initially used the infected culture supernatant (serum-free) as the test virus, following standard procedures. However, in this case, the IC_99_ of chlorous acid water was 9.9 ppm, about 1/200 as effective compared to using purified virus particles. SDS-PAGE analysis of the culture supernatant revealed proteins believed to be of cellular origin, such as a protein with a molecular weight of approximately 70 kDa (Fig 3A). Moreover, amino acids originally present in the culture medium might have weakened the efficacy of chlorous acid water. In other words, to accurately evaluate the effects of chlorous acid water, it is necessary to use highly purified virus particles.

The inactivation capacity of SARS-CoV-2 was significantly affected by the presence of proteins. With a 0.5% PP load, simulating contamination by vomitus, the IC_99_ increased to 76 ppm (about 1,500 times), and its efficacy was greatly reduced. Under conditions simulating saliva with 5% FBS, the IC_99_ was 11 ppm (about 220 times), while for blood contamination with 0.3% SRBCs + 0.3% BSA, the IC_99_ was 5.3 ppm (about 100 times), and with 0.03% BSA, the IC_99_ was 0.31 ppm (about 6 times). The 0.5% PP load particularly tended to inactivate the effects of chlorous acid water. This difference could be due to the difference in the amount of protein added, but on the other hand, past experiments with an excess of amino acids revealed that viruses were specifically inactivated by cysteine and histidine in chlorous acid water [15], suggesting that the difference may also be due to the types of amino acids on the surfaces of PP, BSA, and SRBCs.

In practical applications, chlorous acid water is generally used at high concentrations, such as 200 ppm, for disinfection. Therefore, even under the most challenging conditions of 0.5% PP load, SARS-CoV-2 can be sufficiently inactivated, and chlorous acid water is believed to possess significant disinfection capacity against the virus.

In this study, chlorous acid water demonstrated high inactivation capacity against SARS-CoV-2, including the Delta and Omicron variants. Furthermore, chlorous acid water has low toxicity, as evidenced by its approval as a food additive by the Ministry of Health, Labor, and Welfare [14], and it is considered safe due to the gradual loss of chlorine ions over a period of several hours [12]. Therefore, the results of this study indicate that chlorous acid water is effective as a disinfectant against SARS-CoV-2 and may be suitable for use in public and medical facilities. Moving forward, it will be necessary to verify the disinfection effect in real-world settings while being mindful of potential issues such as the corrosion of metals [25].

## Supporting information

S1 Table Parameters based on the Chick-Watson model. The results of reagent concentration and viral growth inhibition were fitted to the Chick-Watson model to calculate rate constant (k) and dilution coefficient (n). Values are shown as the form of “average ± standard deviation”. Coefficient of determination (R2) values were also calculated. In the case of a one-minute reaction, it is indicated in the table.
(DOCX)

S2 Table Number of apparently intact and destroyed particles by electron microscopy of viruses concentrated by PEG precipitation and ultracentrifugation. (DOCX)

S1 Raw images The original images of Fig 3A and 3B, and the source images for counting in Fig 3C. (PDF)
