# Supplementary material for: Virus purification highlights the high susceptibility of SARS-CoV-2 to a chlorine-based disinfectant, chlorous acid
Source: PLoS One. 2023 Jul 14;18(7):e0288634. doi: 10.1371/journal.pone.0288634 (PMC10348549; doi:10.1371/journal.pone.0288634)
Supplement: S1 Table — The results of reagent concentration and viral growth inhibition were fitted to the Chick-Watson model to calculate rate constant (k) and dilution coefficient (n). Values are shown as the form of “average ± standard deviation”. Coefficient of determination (R2) values were also calculated. In the case of a one-minute reaction, it is indicated in the table. (DOCX) [file pone.0288634.s001.docx]

Supplementary Table 1. Parameters based on the Chick-Watson model.

| Virus | Reagent [Reaction time] | k | n | R^2^ |
| --- | --- | --- | --- | --- |
| D614G | Chlorous acid water [10 min] | 0.019 ± 0.001 | 1.0 ± 0.02 | 1.00 |
| Delta | Chlorous acid water [10 min] | 0.091 ± 0.033 | 0.34 ± 0.08 | 0.89 |
| Omicron | Chlorous acid water [10 min] | 0.066 ± 0.021 | 0.67 ± 0.11 | 0.96 |
| D614G, PEG-purified, 1/10-diluted | Chlorous acid water [1 min] | 5.3 ± 0.5 | 1.1 ± 0.2 | 0.97 |
| D614G, Ultracentrifugation-purified | Chlorous acid water [1 min] | 2.3 ± 0.3 | 0.49 ± 0.08 | 0.95 |
| D614G, PEG-purified, 1/10-diluted | Chlorous acid water + 0.03% BSA [10 min] | 0.24 ± 0.04 | 0.17 ± 0.05 | 0.74 |
| D614G, PEG-purified, 1/10-diluted | Chlorous acid water + 0.3% SRBCs, 0.3% BSA [10 min] | 0.12 ± 0.09 | 0.35 ± 0.22 | 0.67 |
| D614G, PEG-purified, 1/10-diluted | Chlorous acid water + 0.5% PP [10 min] | 0.00060 ± 0.00039 | 1.3 ± 0.1 | 0.98 |
| D614G, PEG-purified, 1/10-diluted | Chlorous acid water + 5% FBS [10 min] | 1.1 ± 0.2 | 0.26 ± 0.05 | 0.92 |
| D614G, PEG-purified, 1/10-diluted | Sodium hypochlorite solution [1 min] | 2.3 ± 0.2 | 0.21 ± 0.05 | 0.82 |

The results of reagent concentration and viral growth inhibition were fitted to the Chick-Watson model to calculate rate constant (k) and dilution coefficient (n). Values are shown as the form of “average ± standard deviation”. Coefficient of determination (R^2^) values were also calculated. In the case of a one-minute reaction, it is indicated in the table.
