# Supplementary material for: Virus purification highlights the high susceptibility of SARS-CoV-2 to a chlorine-based disinfectant, chlorous acid
Source: PLoS One. 2023 Jul 14;18(7):e0288634. doi: 10.1371/journal.pone.0288634 (PMC10348549; doi:10.1371/journal.pone.0288634)
Supplement: S2 Table — (DOCX) [file pone.0288634.s002.docx]

Supplementary Table 2. Number of apparently intact and destroyed particles by electron microscopy of viruses concentrated by PEG precipitation and ultracentrifugation.

| Field # | PEG precipitation | | Ultracentrifugation | |
| --- | --- | --- | --- | --- |
|  | Normal | Destroyed | Normal | Destroyed |
| 1 | 34 | 0 | 16 | 4 |
| 2 | 23 | 2 | 8 | 18 |
| 3 | 9 | 0 | 9 | 9 |
| 4 | 83 | 2 | 3 | 13 |
| 5 | 33 | 2 | 8 | 1 |
| Sum | 182 | 6 | 44 | 45 |
| Total | 188 | | 89 | |
| % | 96.8 | 3.2 | 49.4 | 50.6 |
