# Supplementary material for: Virus purification highlights the high susceptibility of SARS-CoV-2 to a chlorine-based disinfectant, chlorous acid
Source: PLoS One. 2023 Jul 14;18(7):e0288634. doi: 10.1371/journal.pone.0288634 (PMC10348549; doi:10.1371/journal.pone.0288634)
Supplement: S1 Raw images — (PDF) [file pone.0288634.s003.pdf]

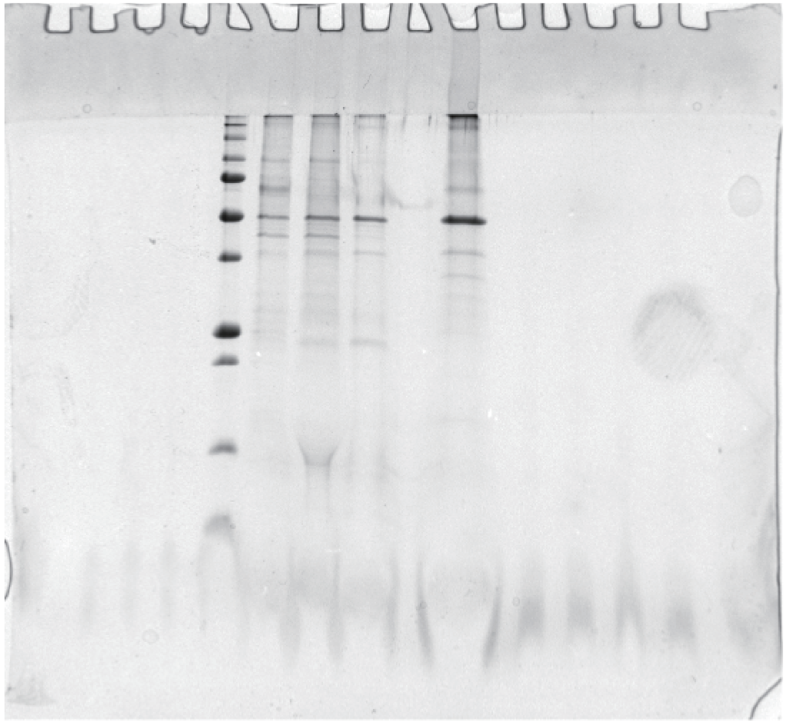

The original image of Fig 3A

An equivalent quantity of purified viruses was analyzed using SDS-PAGE and stained with Coomassie Brilliant Blue R-250. The gel image was then captured using the iBright 1500 system (Invitrogen).

PEG precipitation

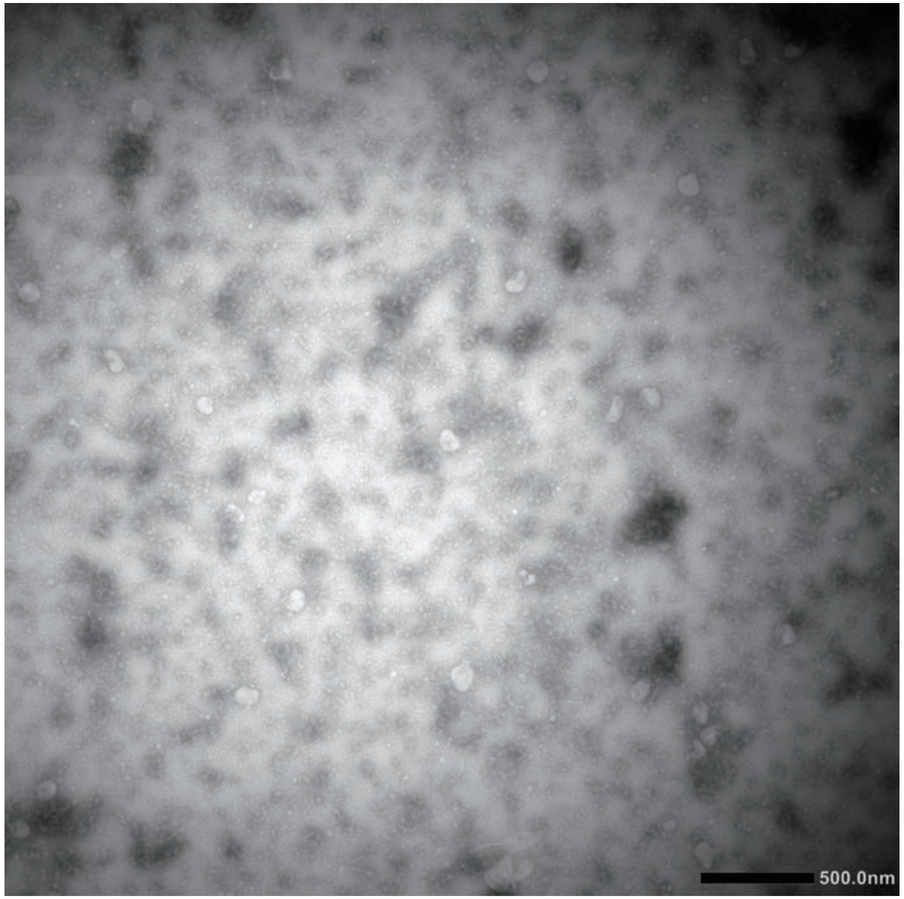

Ultracentrigation

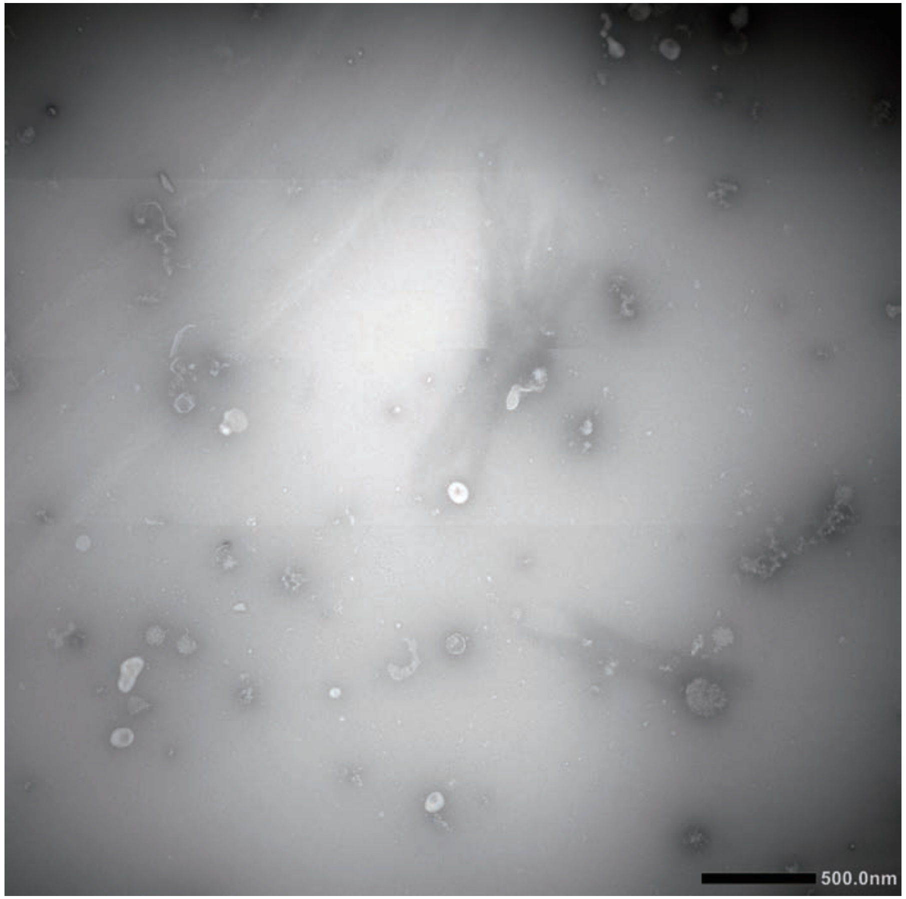

<u>The original images of Fig 3B.</u>

The photographs were taken by using a transmission electron microscope (JEM-1400, JEOL, Tokyo, Japan) at 80 kV.

Images were recorded with an equipped CCD camera (1024 x 1024 pixels).

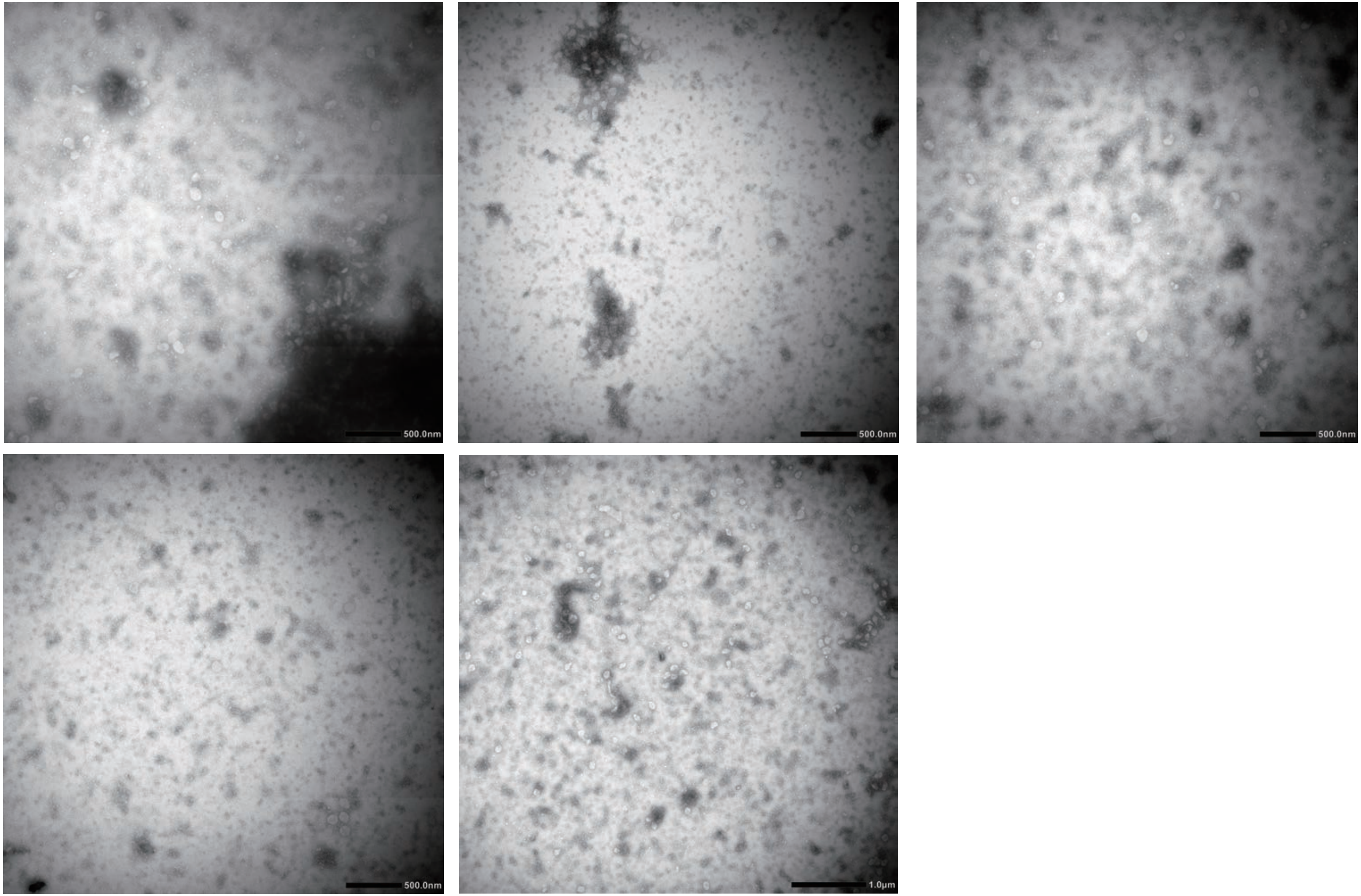

The source images for counting in Fig 3C (PEG precipitation).

The photographs were taken by using a transmission electron microscope (JEM-1400, JEOL, Tokyo, Japan) at 80 kV.

Images were recorded with an equipped CCD camera (1024 x 1024 pixels).

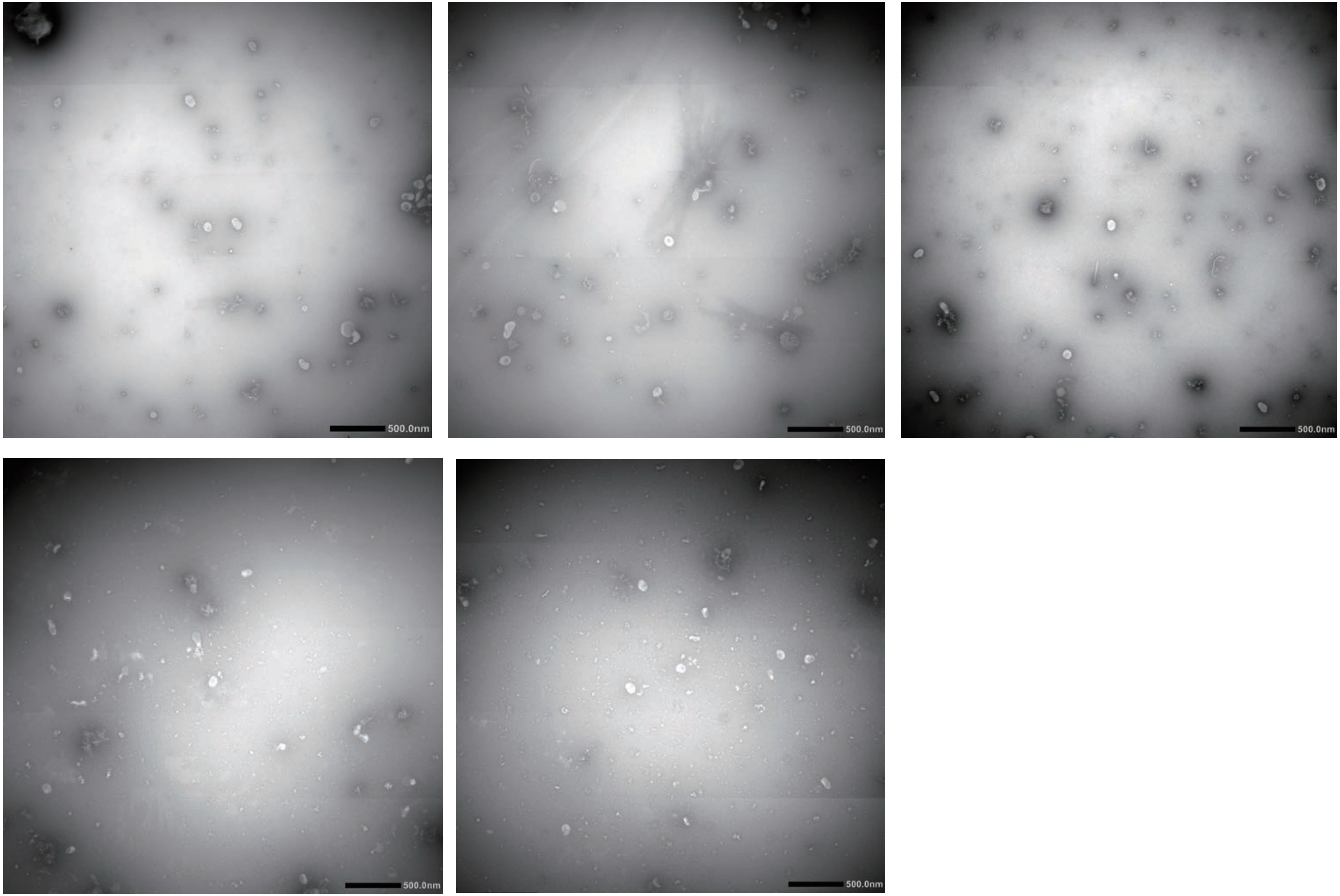

The source images for counting in Fig 3C (Ultracentrifugation).

The photographs were taken by using a transmission electron microscope (JEM-1400, JEOL, Tokyo, Japan) at 80 kV.

Images were recorded with an equipped CCD camera (1024 x 1024 pixels).
